# AAV13 Enables Precise Targeting of Local Neural Populations

**DOI:** 10.3390/ijms232112806

**Published:** 2022-10-24

**Authors:** Zengpeng Han, Nengsong Luo, Yang Wu, Jiaxin Kou, Wenyu Ma, Xin Yang, Yuxiang Cai, Lin Ma, Lu Han, Xiujie Wang, Hualing Qin, Qing Shi, Jie Wang, Chaohui Ye, Kunzhang Lin, Fuqiang Xu

**Affiliations:** 1 State Key Laboratory of Magnetic Resonance and Atomic and Molecular Physics, Key Laboratory of Magnetic Resonance in Biological Systems, Wuhan Center for Magnetic Resonance, Innovation Academy for Precision Measurement Science and Technology, Chinese Academy of Sciences, Wuhan 430071, China; 2 University of Chinese Academy of Sciences, Beijing 100049, China; 3 Shenzhen Key Laboratory of Viral Vectors for Biomedicine, Key Laboratory of Quality Control Technology for Virus-Based Therapeutics, Guangdong Provincial Medical Products Administration, NMPA Key Laboratory for Research and Evaluation of Viral Vector Technology in Cell and Gene Therapy Medicinal Products, The Brain Cognition and Brain Disease Institute (BCBDI), Shenzhen Institute of Advanced Technology, Chinese Academy of Sciences, Shenzhen-Hong Kong Institute of Brain Science-Shenzhen Fundamental Research Institutions, Shenzhen 518055, China; 4 Wuhan National Laboratory for Optoelectronics, Huazhong University of Science and Technology, Wuhan 430074, China; 5 Tongji Medical College, Huazhong University of Science and Technology, Wuhan 430030, China; 6 College of Life Sciences, Wuhan University, Wuhan 430072, China; 7 Center for Excellence in Brain Science and Intelligence Technology, Chinese Academy of Sciences, Shanghai 200031, China

**Keywords:** adeno-associated virus 13, confined diffusion, local gene delivery, small nucleus targeting, sparse labeling, functional monitoring

## Abstract

As powerful tools for local gene delivery, adeno-associated viruses (AAVs) are widely used for neural circuit studies and therapeutical purposes. However, most of them have the characteristics of large diffusion range and retrograde labeling, which may result in off-target transduction during in vivo application. Here, in order to achieve precise gene delivery, we screened AAV serotypes that have not been commonly used as gene vectors and found that AAV13 can precisely transduce local neurons in the brain, with a smaller diffusion range than AAV2 and rigorous anterograde labeling. Then, AAV13-based single-viral and dual-viral strategies for sparse labeling of local neurons in the brains of C57BL/6 or Cre transgenic mice were developed. Additionally, through the neurobehavioral test in the ventral tegmental area, we demonstrated that AAV13 was validated for functional monitoring by means of carrying Cre recombinase to drive the expression of Cre-dependent calcium-sensitive indicator. In summary, our study provides AAV13-based toolkits for precise local gene delivery, which can be used for in situ small nuclei targeting, sparse labeling and functional monitoring.

## 1. Introduction

Gene-drug delivery to specific tissues can be achieved through non-viral vectors or viral vectors [1]. Non-viral vectors have attracted much attention due to their amenability to surface modifications, ease of synthesis and low immunogenicity, but they have a low transportation efficiency [2,3]. Currently, the commonly used viral vectors are adeno-associated viruses (AAVs), because they show low immunogenicity and high transduction efficiency and can mediate long-term gene expression [4,5]. AAVs can be used for the targeting and manipulating of specific cell types within the nervous system; they have become powerful local gene delivery tools for understanding the roles of specific neural cells and brain nuclei as well as gene therapy for brain diseases [6]. CNS-directed AAV gene therapies produce promising pre-clinical outcomes, especially when offered early in the course of the disease, and are increasingly being translated into clinical trials [7]. The mechanism of AAV transduction is via the interaction of AAV capsid with the cell surface receptor, and the extent of viral spread and cell tropism could be quite different between serotypes depending on the target tissue and species; therefore, it is advisable to use suitable AAV serotypes for transduction depending on the needs of each experiment [7,8,9,10].

During direct intraparenchymal administration, accurate local labeling or gene-drug delivery to small nuclei of the brain requires AAV vectors that have a confined diffusion range, which characterizes how far the vector can spread from the injection site. AAV1, AAV2, AAV5, AAV8, AAV9 and the engineered variant AAV-DJ are commonly used to target local populations of neurons; among them, AAV1 and AAV2 diffusion are more confined, therefore these capsids are often chosen for applications that require precise targeting [6,11]. However, they can infect non-target brain regions through in situ diffusion, retrograde infection or trans-synaptic transmission [11,12,13,14]. Therefore, in order to achieve precise local gene delivery, it is extremely necessary to develop a tool virus with small diffusion range and rigorous anterograde labeling.

We previously established a single baculovirus–insect cell system for large-scale virus production (OneBac system) and successfully used this system for the preparation of AAV1-13 serotypes [15]. To find AAV serotypes more suitable for precise in situ targeting, we screened AAV serotypes that were not commonly used for gene delivery purposes and found that AAV13 exhibited confined diffusion without retrograde infection and trans-synaptic transmission in the brain, and that its local diffusion range was smaller than AAV2. Furthermore, AAV13 can be used in combination with the Cre-lox system to mediate high-level gene expression for sparse labeling of local neurons and functional monitoring of neuronal activities.

## 2. Results

### 2.1. AAV13 Vector Is Easy to Manufacture

**Figure 1:**
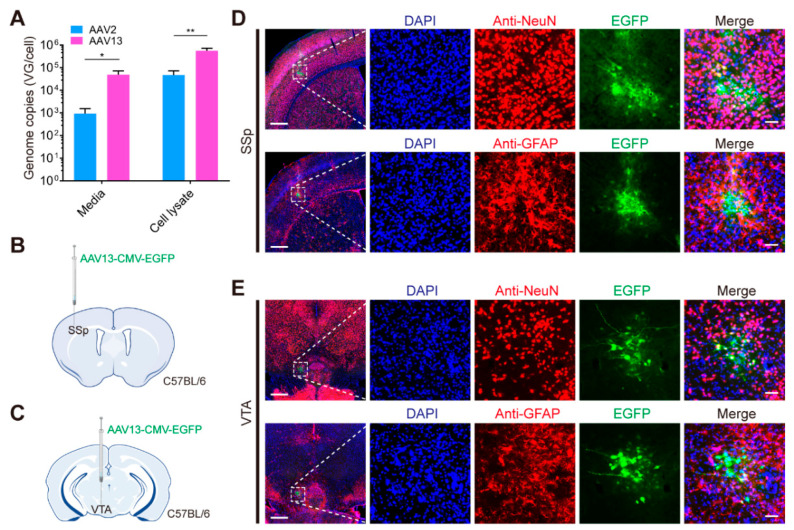
AAV13 vector is easy to manufacture, effectively infects neurons and has a limited spread. (**A**) AAV13 produces higher yields than AAV2. Cell lysate and media were titered and calculated for average yield per cell, respectively. Statistical values are indicated as mean ± SEM (*n* = 4/group). Significant differences are expressed by the *p* value. *: *p* < 0.05; **: *p* < 0.01. (**B**) Schematic diagram of virus injection. AAV13 (2 × 10^9^ vector genomes (VG)) was injected into primary somatosensory area (SSp) of C57BL/6 mice. (**C**) Schematic diagram of virus injection. AAV13 (2 × 10^9^ VG) was injected into ventral tegmental area (VTA) of C57BL/6 mice. (**D**) Immunohistochemistry of cell types by anti-NeuN or anti-GFAP and the fluorescence distribution of EGFP at the injection site of SSp. Scale bar = 500 μm (left 2 panels), 50 μm (right 8 panels). (**E**) Immunohistochemistry of cell types by anti-NeuN or anti-GFAP and the fluorescence distribution of EGFP at the injection site of VTA. Scale bar = 500 μm (left 2 panels), 50 μm (right 8 panels).

For the practical production of AAV13 vectors based on the conventional triple-transfection method, the packing plasmid pAV2/13 was constructed. Subsequently, the efficiency of AAV13 vehicle packaging was assessed. Real-time quantitative PCR (qPCR) assays were performed on media and cellular lysates of HEK293T cells subjected to triple-transfection of packaging plasmids for AAV13 or AAV2. We found that the pAAV2/13 plasmid could be used to package high-titer AAV13 and the yields of viral particles were significantly higher than that of AAV2. Furthermore, significantly more viral particles of AAV13 were released into the medium during virus preparation than those of AAV2 (Figure 1A).

### 2.2. AAV13 Effectively Infects Neurons and Has a Limited Spread

To assess the transduction effect of AAV13, AAV13-CMV-EGFP virus was injected into the primary somatosensory area (SSp) and ventral tegmental area (VTA) of adult mice via stereotactic injection. We found a very limited diffusion of EGFP signals in situ in both SSp and VTA (Figure 1B–E), then the cell types were identified via immunohistochemistry using glial fibrillary acidic protein (GFAP) and neuronal nuclei (NeuN) antibodies, respectively. The nuclei of all the slices were stained blue with 4′,6-diamidino-2-phenylindole (DAPI). Our results show that the vast majority of EGFP-positive cells were neurons, and a very small number of them were astrocytes. These results suggest that AAV13 may be suitable for precise labeling, especially for targeting small nuclei in the brain.

### 2.3. AAV13 Is Suitable for Small Nucleus Labeling

**Figure 2:**
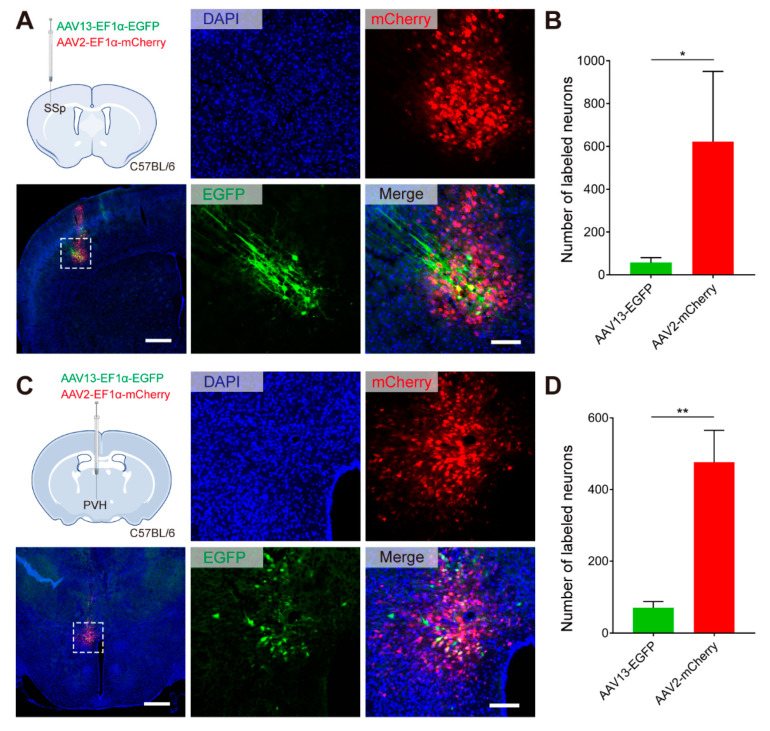
AAV13′s transduction via intraparenchymal administration is more confined than AAV2. (**A**) Comparison of the local transduction effect between AAV13 and AAV2 through fluorescence distribution of EGFP (AAV13) and mCherry (AAV2) at the SSp. AAV13-EF1α-EGFP and AAV2-EF1α-mCherry viruses were mixed at a particle ratio of 1:1 (2 × 10^9^ VG in total) and injected into SSp of C57BL/6 mice. Scale bar = 500 μm (left 1 panel), 100 μm (right 4 panels). (**B**) Quantification of AAV13- and AAV2 -infected neurons in SSp. Statistical values are presented as mean ± SEM (*n* = 3 animals). Significant differences are expressed by the *p* value. *: *p* < 0.05. (**C**) Comparison of the local transduction effect between AAV13 and AAV2 through fluorescence distribution of EGFP (AAV13) and mCherry (AAV2) at the PVH. AAV13-EF1α-EGFP and AAV2-EF1α-mCherry viruses were mixed at a particle ratio of 1:1 (1.5 × 10^9^ VG in total) and injected into PVH of C57BL/6 mice. Scale bar = 500 μm (left 1 panel), 100 μm (right 4 panels). (**D**) Quantification of AAV13- and AAV2-infected neurons in PVH. Statistical values are presented as mean ± SEM (*n* = 3 animals). Significant differences are expressed by the *p* value. **: *p* < 0.01.

As an effective local targeting vehicle, AAV2 is widely used in the analysis and manipulation of different types of neural circuits [16,17,18] as well as therapeutic applications [19]. To assess the performance of AAV13 in situ tagging, AAV13-EF1α-EGFP and AAV2-EF1α-mCherry were mixed in equal amounts and injected into the SSp (Figure 2A) and the paraventricular hypothalamic nucleus (PVH) (Figure 2C). In the SSp, only small clusters of green-fluorescence-positive neurons could be observed, and in the PVH, the small nuclei above the hypothalamus, fluorescent expression exhibited the same characteristics. Quantitative analysis found that AAV13-positive cells were significantly less in number than AAV2-positive cells in both SSp (Figure 2B, *p* = 0.0139) and PVH (Figure 2D, *p* = 0.0015). In general, AAV13 infection exhibits a smaller diffusion range than AAV2.

Furthermore, to further verify the propagation characteristics of AAV13, AAV13-hSyn-Cre was injected into SSp of Ai14 (CAG promoter-driven and Cre-dependent expression of tdTomato reporter) mice [20] (Figure S1). As a biocatalyst, a small amount of Cre recombinase can initiate fluorescent expression, so the use of Ai14 mice can more sensitively detect the spread of AAV13. After 3 weeks post-injection, confined tdTomato fluorescence signals were detected at the injection site, but no tdTomato-expressing cell bodies were observed in the other regions, which confirms that AAV13 permits rigorous anterograde transduction without the features of retrograde infection and trans-synaptic labeling.

### 2.4. AAV13 Permits Precise Sparse Labeling

**Figure 3:**
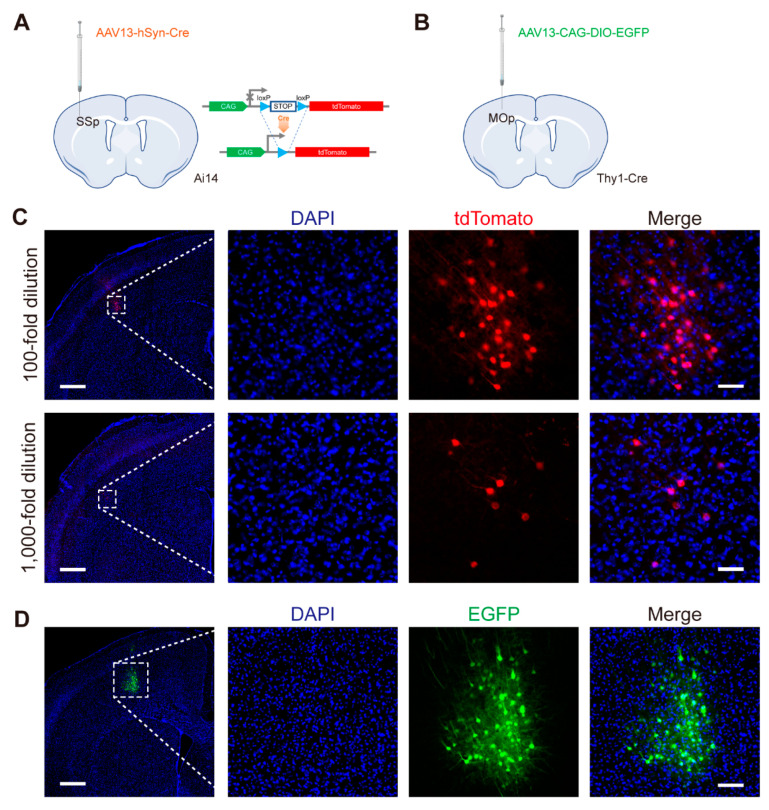
Single-viral vector sparse labeling strategy based on AAV13. (**A**) Schematic diagram of virus injection in SSp. Different concentrations of AAV13-hSyn-Cre (2 × 10^7^ VG and 2 × 10^6^ VG, respectively) were injected into SSp region of Ai14 transgenic mice, in which the expression of tdTomato fluorescent reporter is Cre-dependent. (**B**) Schematic diagram of virus injection in MOp. AAV13-CAG-DIO-EGFP (2 × 10^9^ VG) was injected into primary motor area (MOp) of Thy1-Cre mice. DIO, double-floxed inverted orientation. (**C**) AAV13-hSyn-Cre mediated neuronal sparse labeling in SSp brain region of Ai14 mice. 100-fold dilution, 2 × 10^7^ VG; 1,000-fold dilution, 2 × 10^6^ VG. Scale bar = 500 μm (left 2 panels), 50 μm (right 6 panels). (**D**) AAV13-CAG-DIO-EGFP mediated neuronal sparse labeling in MOp brain region of Thy1-Cre mice. Scale bar = 500 μm (left 1 panel), 100 μm (right 3 panels).

Access to individual neurons is essential in studies dissecting the mechanisms encoding complex functions in the nervous system; sparse labeling is one of the main strategies to resolve individual nerve cells or their precursors [21]. Localized spread of AAV13 itself has the potential to achieve a sparse effect. Based on a single-viral vector strategy, we validated that AAV13 enables sparse labeling. By diluting the virus, 100-fold and 1,000-fold-diluted AAV13-hSyn-Cre were injected into the SSp of Ai14 transgenic mice, shown in Figure 3A,C, respectively. AAV13 led to tdTomato expression in a relatively small zone around the injection site, and in the case of 1,000-fold dilution, the number of fluorescence signals was sparse to less than ten neurons (Figure 3C). Combined with Thy1-Cre transgenic mice, AAV13-CAG-DIO-EGFP, likewise, exhibited sparsely site-localized labeling in the primary motor area (MOp) (Figure 3D).

**Figure 4:**
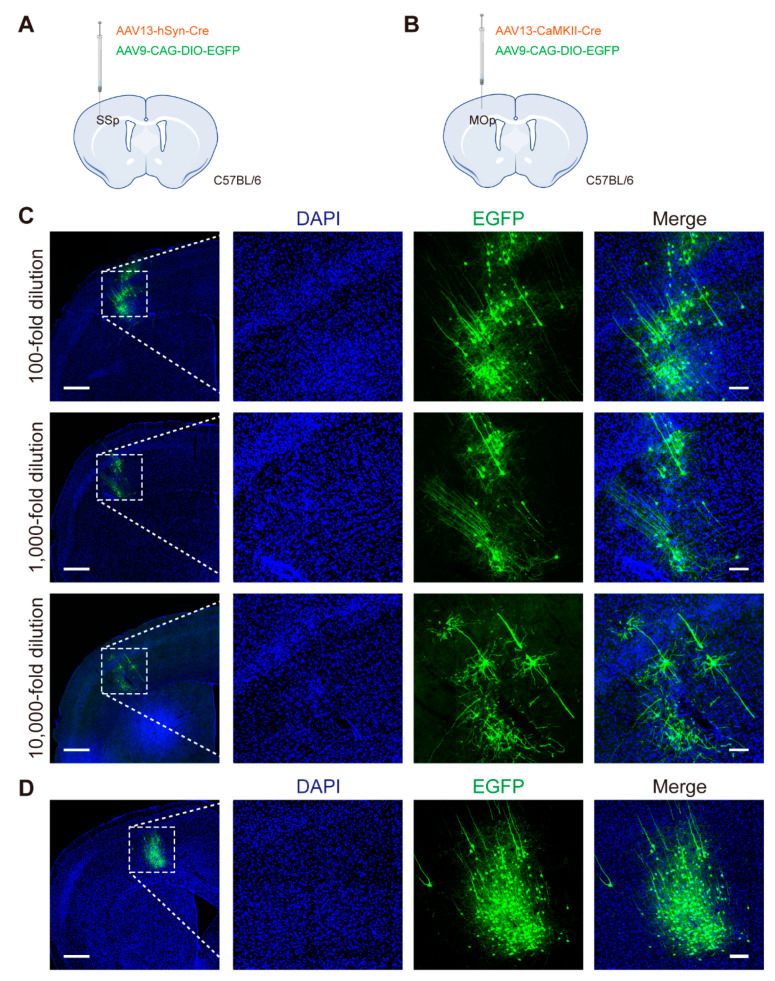
Dual-viral vector sparse labeling strategy based on AAV13. (**A**) Schematic diagram of virus injection in SSp. Different concentrations of AAV13-hSyn-Cre were respectively mixed with AAV9-CAG-DIO-EGFP (2 × 10^9^ VG) and injected into SSp region of C57BL/6 mice. (**B**) Schematic diagram of virus injection into MOp. AAV13-hSyn-Cre (2 × 10^9^ VG) mixed with AAV9-CAG-DIO-EGFP (2 × 10^9^ VG) was injected into MOp region of C57BL/6 mice. (**C**) AAV13-hSyn-Cre combined with AAV9-CAG-DIO-EGFP for neuronal sparse labeling in SSp brain region of C57BL/6 mice. 100-fold dilution, 2 × 10^7^ VG; 1,000-fold dilution, 2 × 10^6^ VG; 10,000-fold dilution, 2 × 10^5^ VG. Scale bar = 500 μm (left 3 panels), 100 μm (right 9 panels). (**D**) AAV13-CaMKII-Cre combined with AAV9-CAG-DIO-EGFP for cell-type specific neuronal sparse labeling in MOp brain region of C57BL/6 mice. Scale bar = 500 μm (left 1 panel), 100 μm (right 3 panels).

To clearly label the morphology of single neurons, the fluorescence expression effect must be strong while being sparse; thus, we also designed a dual-viral vector strategy. As a widely used CNS transduction vector, AAV9 exhibits efficient infection of neurons and high abundance of gene expression, so AAV13-hSyn-Cre as a “controller” mixed with AAV9-CAG-DIO-EGFP (“reporter” or “amplifier”) were injected into the SSp regions of C57BL/6 mice (Figure 4A,C). The “controller” AAV13-hSyn-Cre limited the fluorescence-positive neurons to a small zone; by adjusting its titer, the number of labeled neurons could be sparse. When AAV13-hSyn-Cre was diluted 10,000-fold, the number of labeled neurons was less than ten, and fluorescently highlighted expression of Cre-dependent AAV9-CAG-DIO-EGFP enabled clear visualization of neuronal axonal morphology (Figure 4C). Similarly, AAV13 can carry the glutamatergic-specific CaMKII promoter to achieve dual-viral vector strategy for sparsely highlighted labeling of cell-type specific neurons in the MOp brain region (Figure 4B,D).

### 2.5. Use of AAV13 for Functional Circuit Interrogation

**Figure 5:**
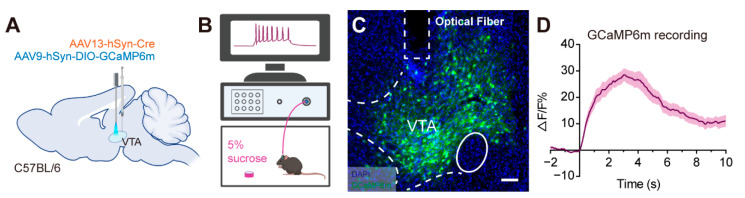
AAV13 can be used for functional circuit interrogation. (**A**) Schematic diagram of AAV injections and fiber implantations for monitoring neural activities of VTA. AAV13-hSyn-Cre mixed with AAV9-hSyn-DIO-GCaMP6m (3 × 10^9^ VG in total) was injected into VTA. An optical fiber was implanted into VTA for the detection of calcium transients. (**B**) Schematic diagram of calcium transient detection in mice under the reward behavior tasks. (**C**) The GCaMP6m expression in VTA. Scale bar = 100 μm. (**D**) Average ΔF/F signals were recorded during the behavior tasks.

The ventral tegmental area (VTA) is a hub for mesocorticolic circuits that plays significant roles in reward, motivation, cognition and aversion [22]. To study the application of AAV13 in the dissection of functional circuits, we injected AAV13-Cre with the Cre-inducible vector AAV9-DIO-GCaMP6m into the VTA (Figure 5A). Sucrose solution was put into the behavior box and served as a reward for the mice (Figure 5B). As expected, VTA neurons were labeled (Figure 5C) and an increase in calcium signals was recorded while the mice licked sugar water (Figure 5D). These results indicate that AAV13 permits functional circuit interrogation.

## 3. Discussion

**Figure 6:**
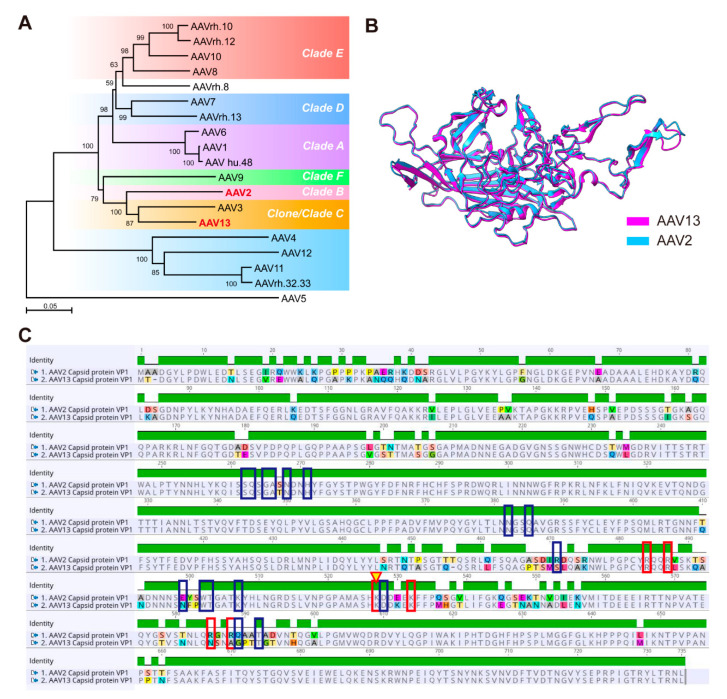
Phylogenetic identity and capsid protein analysis of AAV13. (**A**) Cladogram demonstrating the categorization of AAV13 in the clades proposed by Gao et al. [23]. (**B**) Structural comparison of AAV13 and AAV2. Structural information was obtained from Protein Data Bank (PDB) (AAV13, 7L6H; AAV2, 6IH9) and the figure was generated using UCSF ChimeraX. (**C**) VP1 amino acid sequence alignment between AAV13 and AAV2. Red boxes indicate key sites for AAV2 binding to HSPG, yellow triangles indicate key residues for AAV13 binding to HSPG, dark blue boxes indicate key residues for AAV2 binding to AAVR.

Multiple AAV serotypes have been isolated from tissue culture stocks and humans, as well as non-human primates [9,23,24,25]. Schmidt et al. originally isolated AAV13, named it AAV.VR942 and identified that its transduction activity requires cell surface heparan sulfate proteoglycans (HSPG) [26]. Mietzsch et al. determined the capsid structures of AAV13 via cryo-electron microscopy and three-dimensional image reconstruction to 2.76 Å resolution, demonstrating that AAV13 is structurally slightly more similar to AAV2 (96%), while phylogenetic analysis revealed the closest related AAV serotype to be AAV3 [27] (Figure 6A,B). AAV13 has not yet been used as a gene delivery vehicle for gene therapy or neuroscience research, thus we established a convenient preparation system for AAV13, interrogated the infectious properties of AAV13 in the brain and found its transduction properties of confined diffusion.

Cladogram analysis for the AAV serotypes showed that AAV13 should belong to clade C [23,26,27] (Figure 6A). Common to AAV2 and AAV3, AAV13 can bind to HSPG [26,28,29,30] and to the A20 antibody [31]. For AAV13, the critical VP protein residue for HSPG binding is K528, different from AAV2, in which those residues are R585 and R588 [26] (Figure 6C). AAV’s infection starts with attachment to serotype-specific glycan primary receptors (e.g., HSPG), followed by co-receptor-mediated endocytotic entry; the cellular protein AAVR was implicated as an essential receptor for entry across a panel of AAV serotypes into representative cell types and, in vivo, in mice [32,33]. Sixteen residues on AAV2 capsid proteins were identified as contacts with the AAVR PKD1 domain [34], and three on AAV13 differed accordingly (471, 499, 589) (Figure 6C). A basic fact is that AAV13 has smaller diffusion range and lower-intensity fluorescence expression than AAV2 (Figure 2). Inferentially, strong interaction with attachment factor may limit AAV13 particle diffusion, and weak binding to entry factor or ubiquitinated proteasome-mediated degradation [35] may affect gene expression intensity. A more confined spread may suggest that AAV13 has other possible attachment factors distinct from HSPG.

Replacing surface-exposed tyrosines of AAV2 and AAV2 mutant capsid with phenylalanine (YF mutation) to reduce virion degradation has been demonstrated as an effective means of improving transgene expression efficiency [36,37]. AAV2-7m8 was developed by inserting the heptamer peptide (LGETTRP) into loop IV of AAV2, allowing enhanced retinal targeting over its parental serotype AAV2 [38,39]. While 7m8 insertion led to higher-intensity gene expression, the spread of gene expression remained unchanged compared to the parental serotypes [39]. Given the strong structural similarity between AAV13 and AAV2, the YF mutation or 7m8 modification of AAV13 may be a valid test to improve its transduction efficiency.

For sparse labeling, the smaller diffusion range of AAV13, as a “controller”, is an advantage to achieve sparseness, and the confined diffusion leads to labeling in a relatively small area (Figure 3 and Figure 4). To obtain a finer strong labeling, an approach to give the “amplifier” (e.g., AAV9-DIO-EGFP mentioned above) stronger expression is needed. Combined with the fluorescence micro-optical sectioning tomography (fMOST) platform [40], AAV13-based and Cre-dependent sparse labeling of local neurons will be applicable to whole-brain reconstruction that can more precisely study the complete morphologies of individual neurons at the whole-brain level [41].

As a tool to facilitate in situ gene delivery of small nuclei, AAV13 has stringent anterograde infection (Figure S1) and is validated for functional monitoring (Figure 5). Our research tentatively explores the instrumentalization of AAV13, a process that will be advanced by the more mechanistic study of its properties.

## 4. Materials and Methods

### 4.1. Plasmid Construction

The AAV13 *Cap* gene sequence was retrieved from Genbank (accession number: EU285562) and synthesized on the backbone plasmid as a template, after PCR amplification using primer C13F (5′- atgatttaaatcaggtatgactgacggttaccttcca-3′) and C13R (5′-tcaaccggtttattgattaacacgtaattacagattacgagtcaggtatctggtgc-3′). The AAV13 *Cap* fragment replaced the AAV1 *Cap* fragment on pAAV2/1 (Addgene, Watertown, MA, USA, 112862) through SwaI and AgeI restriction endonuclease, followed by transformation into chemically competent *E. coli* stain Stbl3. The positive clone was picked after PCR identification and the plasmid was extracted to obtain pAAV-RC2/13 packaging plasmid.

### 4.2. AAV Vector Manufacturing

HEK293T cells were obtained from the American Type Culture Collection (Manassas, VA, USA) and maintained in suspension culture in Balance CD (Chuangling Cell-wise, Shanghai, China, CW01001) medium supplemented with 1% penicillin/streptomycin (BasalMedia, Shanghai, China, S110JV) at 37 °C and 5% CO_2_. Sixteen hours before transfection, cells were transferred to adherent cultured in DMEM (BasalMedia, L110KJ) with 2% fetal bovine serum (Thermo Fisher Scientific GIBCO, Waltham, MA, USA, 10099141C) and 1% penicillin/streptomycin (BasalMedia, Shanghai, China, S110JV). Briefly, AAV vectors were produced by triple plasmid transient transfection with linear polyethylenimine (Polysciences, Warrington, PA, USA, 24765-1), viral particles were harvested at 72 h post-transfection and purified via iodixanol gradient ultracentrifugation [42], and buffer exchange was performed by using phosphate buffered saline (PBS) with 0.001% Pluronic F68 (Thermo Fisher Scientific, Waltham, MA, USA, 24040032) via Amicon filtration (Merck Millipore, Billerica, MA, USA, UFC910024). The purified rAAVs were titered via qPCR using the iQ SYBR Green Supermix kit (Bio-Rad, Hercules, CA, USA, 1708884). The obtained virus titers are shown in Supplementary Table S1. All viral vectors were aliquoted and stored at −80 °C until use.

### 4.3. Research Animals

All procedures were approved by the Animal Care and Use Committee of Innovation Academy for Precision Measurement Science and Technology, Chinese Academy of Sciences (approval No. APM20026A) in 12 December 2020. Adult (8–10 weeks old) C57BL/6 mice (male, *n* = 21, Hunan SJA Laboratory Animal Company, Changsha, Hunan, China), Ai14 transgenic mice (male, *n* = 6, The Jackson Laboratory, Bar Harbor, ME, USA) and Thy1-Cre transgenic mice (male, *n* = 3, provided by Zhejiang University, Hangzhou, Zhejiang, China) were used for the experiments. Mice were randomly assigned to groups of predetermined sample size. No mice were excluded from these analyses.

### 4.4. Stereotaxic AAV Injection

Mice were deeply anesthetized using 1% pentobarbital intraperitoneally (i.p., 50 mg/kg body weight) and placed in a stereotaxic apparatus (RWD, Shenzhen, Guangdong, China). The injection coordinates were selected according to Paxinos and Franklin’s The Mouse Brain in Stereotaxic Coordinates, 4th edition [43]. Four brain regions were used: SSp (relative to bregma: anterior–posterior axis (AP) +0.50 mm, medial–lateral axis (ML) ±3.00 mm, and dorsal–ventral axis (DV) −2.00 mm); MOp (relative to bregma: AP +1.54 mm, ML ±1.60 mm, and DV −1.60 mm); VTA (relative to bregma: AP −3.20 mm, ML ±0.45 mm, and DV −4.30 mm); and PVH (relative to bregma: AP −0.90 mm, ML ±0.20 mm, and DV −4.80 mm). Virus was injected at a rate of 0.03 μL/min using a stereotaxic injector equipped with a pulled glass capillary (Stoelting, Wood Dale, IL, USA, 53311). After the injection was complete, the micropipette was held for an additional 10 min before being withdrawn. Animals were allowed to recover from anesthesia on a heating pad. Three weeks after injection, we sacrificed the mice to collect brain tissue via transcardic infusion of PBS and 4% paraformaldehyde.

### 4.5. Slice Preparation, Immunohistochemistry and Imaging

Brain slice preparation and imaging were completed according to the previously reported methods [5,44]. The brains were soaked overnight in 4% paraformaldehyde solution. After dehydration was completed with 30% sucrose solution, the brain was sectioned with a thickness of 40 μm via microtome (Thermo Fisher Scientific, Waltham, MA, USA), collected in anti-freeze fluid, and stored at −20 °C for further use. For NeuN staining, sections were incubated with rabbit anti-NeuN (1:800, Abcam, Cambridge, MA, USA) primary antibody overnight at 4 °C. After washing 3 times with PBS, the slices were incubated with the secondary antibody Cy3-conjugated goat anti-rabbit immunoglobulin G (IgG) (1:400, The Jackson Laboratory, Bar Harbor, ME, USA) for 1 h at 37 °C. For GFAP staining, the primary antibody was goat anti-GFAP (1:800, Abcam, Cambridge, MA, USA) and the secondary antibody was rabbit anti-goat IgG conjugated with Cy3 (1:400, The Jackson Laboratory, Bar Harbor, ME, USA). After washing with PBS, all the brain slices attached to the microscope slides were counterstained with DAPI (1:4000, Beyotime, Shanghai, China) and sealed with 70% glycerol. Imaging was performed using a Leica TCS SP8 confocal microscope (Leica, Wetzlar, Germany) or an Olympus VS120 virtual microscopy slide scanning system (Olympus, Tokyo, Japan).

### 4.6. GCaMP6m-Based Calcium Imaging In Vivo

AAV13-hSyn-Cre and AAV9-DIO-GCaMP6m were mixed and injected into the VTA. Optical fiber (core diameter: 200 μm, numerical aperture: 0.37, Inper, Hangzhou, Zhejiang, China) was implanted into the VTA. Mice had visually identifiable GCaMP6m-expressing cells in the VTA two weeks after injection. The manipulation of calcium imaging was described in our previous study [44]. The VTA region was activated via sucrose solution reward, and calcium transients were recorded via exciting GCaMP6m at 470 nm using the fiber photometry system (ThinkerTech, Nanjing, Jiangsu, China).

### 4.7. Data Analysis

Calcium recording data were analyzed using MATLAB R2018b (Mathworks, Natic, MA, USA) and GraphPad Prism 7.0 (GraphPad Software, La Jolla, CA, USA). For neuron counting or viral packaging efficiency comparison, statistical analysis was accomplished using an unpaired *t*-test via GraphPad Prism. Phylogenetic analysis of AAV *Cap* genes of various serotypes was achieved using MEGA11 [45] and modified using Adobe Illustrator CC (Adobe Inc., San Jose, CA, USA). The three-dimensional structure alignment of the VP1 protein of AAV13 and AAV2 was presented using UCSF ChimeraX v1.3 [46,47] (UCSF, San Francisco, CA, USA), and the amino acid sequence alignment was presented using Geneious Prime v2021.2 (Biomatters, Auckland, New Zealand).

## Data Availability

All datasets for this study are included in the manuscript and the supplementary files.

## Supplementary Materials

The following supporting information can be downloaded at: https://www.mdpi.com/article/10.3390/ijms232112806/s1.
