# Supplementary material for: AAV13 Enables Precise Targeting of Local Neural Populations
Source: Int J Mol Sci. 2022 Oct 24;23(21):12806. doi: 10.3390/ijms232112806 (PMC9653909; doi:10.3390/ijms232112806)
Supplement: Supplementary file 1 [file ijms-23-12806-s001.zip › ijms-1966332-SI.pdf]

AAV13 Enables Precise Targeting of Local Neural Populations

Zengpeng Han [1,2,3,†], Nengsong Luo [4,†], Yang Wu [1,2], Jiaxin Kou [5], Wenyu Ma [1,2], Xin Yang [1], Yuxiang Cai [4], Lin Ma [5], Lu Han [6], Xiujie Wang [6], Hualing Qin [6], Qing Shi [3], Jie Wang [1,2], Chaohui Ye [1,2], Kunzhang Lin [3,*] and Fuqiang Xu [1,2,3,4,7,*]

* Correspondence: kz.lin@siat.ac.cn (K.L.); fq.xu@siat.ac.cn (F.X.); Tel.: +86-150-7244-8709 (K.L.); +86-139-9550-5336 (F.X.)
† These authors contributed equally to this work.

Supplementary Information

(Contains Supplementary Table S1 and Figure S1 with Legends)

Supplementary Table S1. rAAV viral vectors titers

No.	AAV Vector	Titer (VG/mL)
1	rAAV13-CMV-EGFP-WPRE-pA	$4×10^{12}$
2	rAAV13-EF1α-EGFP-WPRE-pA	$5×10^{12}$
3	rAAV2-EF1α-mCherry-WPRE-pA	$4×10^{12}$
4	rAAV13-hSyn-Cre-WPRE-pA	$4×10^{13}$
5	rAAV13-CAG-DIO-EGFP-WPRE-pA	$2×10^{13}$
6	rAAV13-CaMKII-Cre-WPRE-pA	$3×10^{13}$
7	rAAV9-CAG-DIO-EGFP-WPRE-pA	$8×10^{12}$
8	rAAV9-hSyn-DIO-GCaMP6m-WPRE-pA	$1×10^{13}$

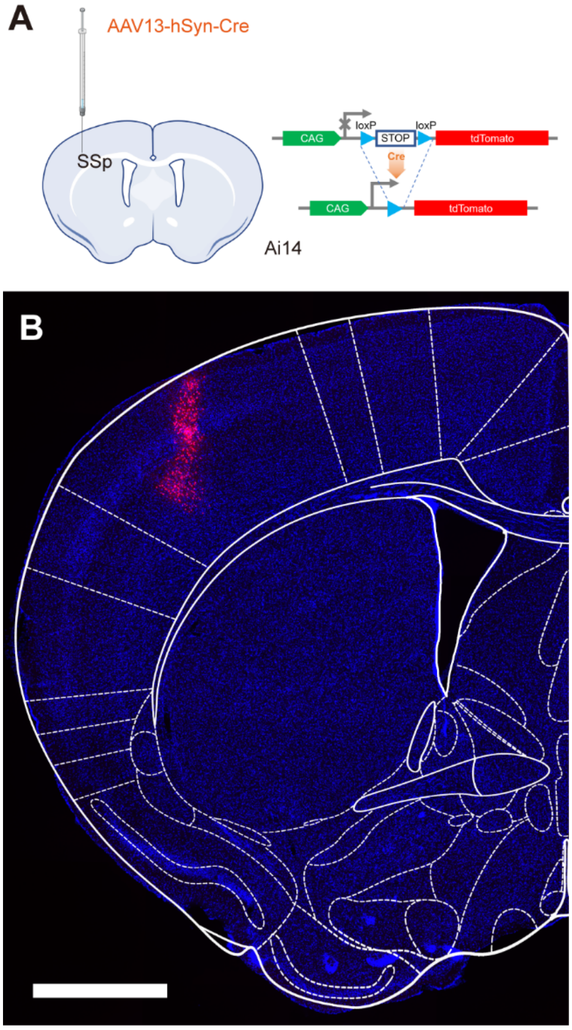

Supplementary Figure S1. AAV13 does not exhibit retrograde or transsynaptic propagation. (**A**) Schematic diagram of virus injection. AAV13-hSyn-Cre ($3 × 10^9$ VG) was injected into SSp region of Ai14 transgenic mice, in which the expression of tdTomato fluorescent reporter is Cre-dependent. (**B**) TdTomato expression was observed only at the injection site. Scale bar = 1 mm.
